# c-Met inhibitors attenuate tumor growth of small cell hypercalcemic ovarian carcinoma (SCCOHT) populations

**DOI:** 10.18632/oncotarget.5151

**Published:** 2015-09-30

**Authors:** Anna Otte, Finn Rauprich, Juliane von der Ohe, Yuanyuan Yang, Friedrich Kommoss, Friedrich Feuerhake, Peter Hillemanns, Ralf Hass

**Affiliations:** ^1^ Biochemistry and Tumor Biology Laboratory, Department of Obstetrics and Gynecology, Hannover Medical School, Hannover, Germany; ^2^ Synlab MVZ Pathologie Mannheim GmbH, Referral Center for Gynecopathology, Mannheim, Germany; ^3^ Institute for Pathology, Hannover Medical School, Hannover, Germany

**Keywords:** SCCOHT, small cell ovarian cancer, c-Met inhibitor, foretinib, tumor growth

## Abstract

A cellular model (SCCOHT-1) of the aggressive small cell hypercalcemic ovarian carcinoma demonstrated constitutive chemokine and growth factor production including HGF. A simultaneous presence of c-Met in 41% SCCOHT-1 cells suggested an autocrine growth mechanism. Expression of c-Met was also observed at low levels in the corresponding BIN-67 cell line (6.5%) and at high levels in ovarian adenocarcinoma cells (NIH:OVCAR-3 (84.4%) and SK-OV-3 (99.3%)). Immunohistochemistry of c-Met expression in SCCOHT tumors revealed a heterogeneous distribution between undetectable levels and 80%. Further characterization of SCCOHT-1 and BIN-67 cells by cell surface markers including CD90 and EpCAM demonstrated similar patterns with differences to the ovarian adenocarcinoma cells. HGF stimulation of SCCOHT-1 cells was associated with c-Met phosphorylation at Tyr^1349^ and downstream Thr^202^/Tyr^204^ phosphorylation of p44/42 MAP kinase. This HGF-induced signaling cascade was abolished by the c-Met inhibitor foretinib. Cell cycle analysis after foretinib treatment demonstrated enhanced G2 accumulation and increasing apoptosis within 72 h. Moreover, the IC_50_ of foretinib revealed 12.4 nM in SCCOHT-1 cells compared to 411 nM and 481 nM in NIH:OVCAR-3 and SK-OV-3 cells, respectively, suggesting potential therapeutic effects. Indeed, SCCOHT-1 and BIN-67 tumor xenografts in NOD^scid^ mice exhibited an approximately 10-fold and 5-fold reduced tumor size following systemic application of foretinib, respectively. Furthermore, foretinib-treated tumors revealed a significantly reduced vascularization and little if any c-Met-mediated signal transduction. Similar findings of reduced proliferative capacity and declined tumor size were observed after siRNA-mediated c-Met knock-down in SCCOHT-1 cells demonstrating that *in vivo* inhibition of these pathways contributed to an attenuation of SCCOHT tumor growth.

## INTRODUCTION

One of the most lethal gynecologic malignancies is caused by ovarian cancer. A variety of different epithelial ovarian cancers have been categorized into two types, whereby type I tumors include low-grade serous, endometrioid, clear cell and mucinous carcinomas which appear clinically indolent. In contrast, type II tumors are characterized by high-grade serous, high-grade endometrioid and undifferentiated carcinomas, as well as malignant mixed mesodermal tumors (carcinosarcomas) with papillary, glandular, and solid patterns displaying highly aggressive cancer cells predominantly observed in advanced tumor stages [1–3]. This differentiated histopathological categorization of ovarian tumor types is also supported by molecular differences. Thus, gene mutations including KRAS, BRAF, ERBB2, PTEN, CTNNB1, and PIK3CA are predominantly detected in type I ovarian tumors. Vice versa, type II tumors often display genetic instabilities with a high frequency of TP53 mutations and cyclin E1 amplifications which directly regulate the proliferative capacity and cell cycle progression [4, 5].

Upon variations of this type of malignant neoplasia, the small cell ovarian carcinoma of the hypercalcemic type (SCCOHT) represents a rare form of an aggressive ovarian tumor which is predominantly observed in young women between ages of 13 to 35. The SCCOHT has a poor prognosis and is associated in most cases with paraendocrine hypercalcemia [6, 7].

Histopathological evaluation of several clinical cases have classified the SCCOHT as a separate pathological entity [6] since this tumor appears different from other ovarian cancer types including ovarian epithelial tumors and ovarian germ cell tumors [8]. However, the etiology of the SCCOHT still remains obscure. Whereas immunohistochemical analysis of the SCCOHT postulated a germ cell-derived tumor [9], other work has also discussed SCCOHT as an epithelial-like originating tumor [7] and genetic analysis of SCCOHT tumor specimen have documented an inhomogeneous tumor entity [10–12]. Genome sequencing of SCCOHT tumor biopsies revealed both, germline and somatic mutations of the *SMARCA4* gene including a stop codon mutation p.Arg1077* and a frameshift p.Pro1180fs [13]. The SMARCA4 gene encodes the transcription activator BRG1 which represents an ATP-dependent helicase of the SWI/SNF family and its mutation was suggested as a potential molecular marker for the SCCOHT [14–16].

Cellular models for the SCCOHT are represented by the BIN-67 [17] and the SCCOHT-1 [18] cell lines. In line with the SCCOHT histology, characterization of BIN-67 and SCCOHT-1 tumor cells indicated heterogeneous populations with certain epithelial and mesenchymal properties. Moreover, SCCOHT-1 tumor cells are carrying a defective *SMARCA4* gene with a loss of BRG1 protein expression [19] and likewise, BIN-67 cells demonstrated biallelic deleterious *SMARCA4* gene mutations [15] which confirms the results in SCCOHT patient biopsies. Whereas mutations in the *SMARCA4* gene and the related *SMARCB1* gene also occur in malignant rhabdoid tumors, further similarities by whole exome sequencing suggested SCCOHT as malignant rhabdoid tumor of the ovary [20]. Furthermore, BIN-67 and SCCOHT-1 cells developed appropriate tumors in xenotransplants and exhibited multiple chemotherapeutic resistances by continued tumor growth [21, 22]. Consistently, various resistant effects are also observed in SCCOHT patients and therefore, reasonable approaches for the treatment of this tumor disease remain unknown. It was thus the aim of the present study, to identify a potential molecular target for a growth arrest of these tumor cells by investigating effects of growth factors such as HGF and the related receptor c-Met in SCCOHT-1 cell cultures in comparison to BIN-67 cells and the established human ovarian adenocarcinoma NIH:OVCAR-3 and SK-OV-3 cell line.

## RESULTS

**Figure 1:**
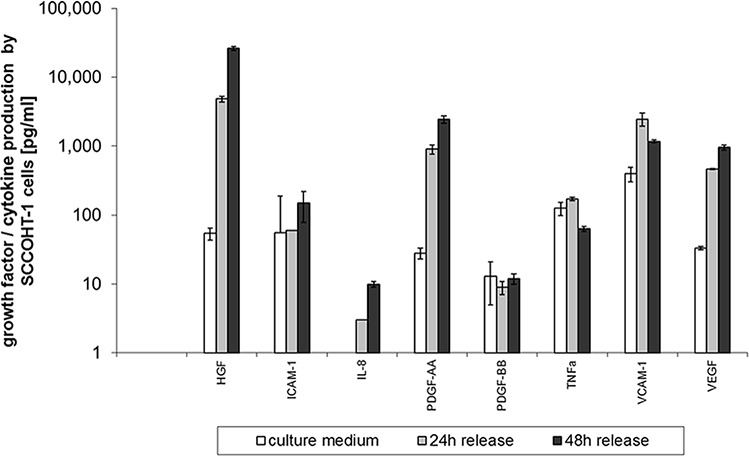
Quantitative production of distinct growth factors and cytokines was measured in supernatants of SCCOHT-1 (2 × 105 cells/ml) after 24 h and 48 h, respectively, using a multiplexed human chemokine assay system Data represent the amount of cytokine/growth factor production [pg/2 × 10^5^ cells] ± s.d. (*n* = 4). (HGF = hepatocyte growth/scatter factor; ICAM-1 = intercellular cell adhesion molecule-1; IL-8 = interleukin-8; PDGF = platelet-derived growth factor; TNFa = tumor necrosis factor-alpha; VCAM-1 = vascular cell adhesion molecule-1; VEGF = vascular endothelial growth factor)

The constitutive production and release of certain cytokines and growth factors by SCCOHT-1 cells was measured in a customized human multiplex ELISA system. Little if any release of ICAM-1, PDGF-BB and TNF-α was detectable in SCCOHT-1 cell culture medium after 24 h and 48 h, respectively. However, there was a significant production of HGF by 4,868 ± 464ng/2 × 10^5^ cells after 24 h which raised to 24,590 ± 1,580ng/2 × 10^5^ cells (*n* = 4) after 48 h (Fig. 1). Moreover, an increase in IL8 production was also paralleled by elevated PDGF-AA levels from 11 ± 2 ng/ml in control medium to 666 ± 100ng/2 × 10^5^ cells after 24 h and 2,167 ± 279ng/2 × 10^5^ cells after 48 h (*n* = 4), respectively. Likewise, release of VCAM-1 and VEGF was significantly elevated by SCCOHT-1 cells (Fig. 1).

**Figure 2:**
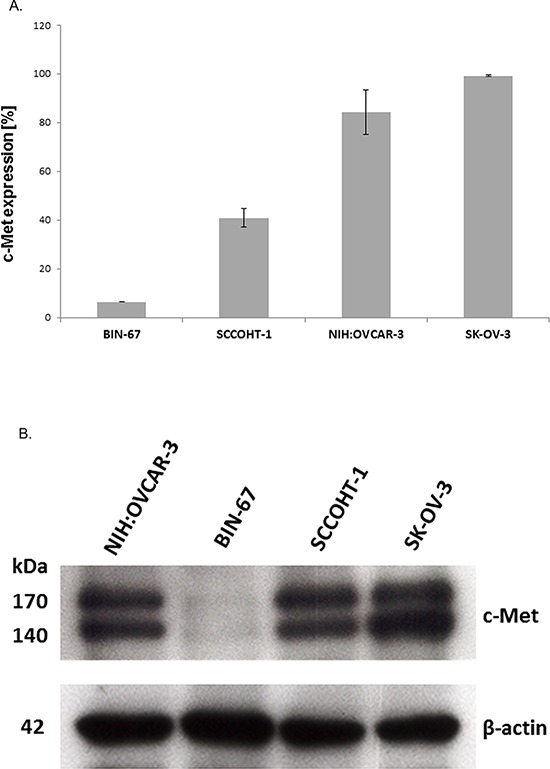
A. Expression of c-Met protein was measured by flow cytometry in BIN-67, SCCOHT-1, NIH:OVCAR-3 and SK-OV-3 cells, respectively Data represent the percentage of c-Met expression ± s.d. (*n* = 4). **B.** Western blot of c-Met expression in the 4 different ovarian cancer cell populations with GAPDH expression as control.

According to the constitutive production and release of HGF by SCCOHT-1 cells, simultaneous expression of the corresponding receptor c-Met was investigated. Analysis by flow cytometry revealed c-Met receptor expression in 6.5 ± 0.1% (*n* = 3) of BIN-67 cells, 40.9 ± 3.8% (*n* = 3) of SCCOHT-1 cells and a majority in ovarian adenocarcinoma cells with 84.4 ± 9.2% (*n* = 3) in NIH:OVCAR-3 cells and 99.3 ± 0.4% (*n* = 3) in SK-OV-3 cells (Fig. 2A). Similar results were obtained by Western blots with the lowest levels of c-Met proteins in BIN-67 cells and high expression levels in NIH:OVCAR-3 cells and SK-OV-3 cells (Fig. 2B).

Although different levels of c-Met expression were observed in SCCOHT-1 and BIN-67 cells, these two populations shared a variety of similar markers and could be distinguished from other ovarian adenocarcinoma cells. Flow cytometry analysis revealed a common expression of surface markers for cell-cell communication including the beta-1 integrin subunit CD29 among all of these ovarian cancer populations which i.e. can function as the α3β1 integrin and may play a role during metastatic diffusion of certain tumor cells. However, marked differences were observed for a variety of other cell surface markers in SCCOHT-1 and BIN-67 cells compared to NIH:OVCAR-3 and SK-OV-3 cells indicating altered tumor cell functionalities. One of these differences included the glycosyl phosphatidyl inositol (GPI)-anchored CD90 antigen expression, also present on some immune cells, hematopoietic stem cells, and a property of mesenchymal stem cells in a normoxic and hypoxic microenvironment [23, 24]. Increased CD90 and vimentin levels were detected during epithelial to mesenchymal transition of non small cell lung cancer (NSCLC) cells [25] and CD90 protein expression by SCCOHT-1 and BIN-67 cells was in contrast to little if any detectable CD90 in NIH:OVCAR-3 and SK-OV-3 cells (Suppl. Fig. S1). Moreover, mesothelin as part of the outer plasma membrane by GPI linkage is overexpressed in several human tumors, including mesothelioma as well as pancreatic and ovarian adenocarcinoma [26] and was detectable in NIH:OVCAR-3 and SK-OV-3 cells in contrast to SCCOHT-1 and BIN-67 cells. In addition, the epithelial cell adhesion molecule (CD326/EpCAM) functions as a transmembrane glycoprotein mediating Ca^2+^-independent homotypic cell-cell adhesion in epithelia and in contrast to an extensive expression in NIH:OVCAR-3 and SK-OV-3 cells this adhesion molecule was undetectable in SCCOHT-1 and BIN-67 cells. Furthermore, the mesenchymal intermediate filament vimentin could be observed in SCCOHT-1, BIN-67 and SK-OV-3 cells and to about 7% NIH:OVCAR-3 cells. In addition, cytokeratins as part of the epithelial intermediate filament proteins were strongly expressed in NIH:OVCAR-3 and SK-OV-3 cells and only to about 27% in SCCOHT-1 and about 2% in BIN-67 cells (Suppl. Fig. S1). Together, these findings suggested differences in cell-cell and cell-matrix interactions between the small cell hypercalcemic ovarian tumor cell types and the ovarian adenocarcinoma cell lines.

**Figure 3:**
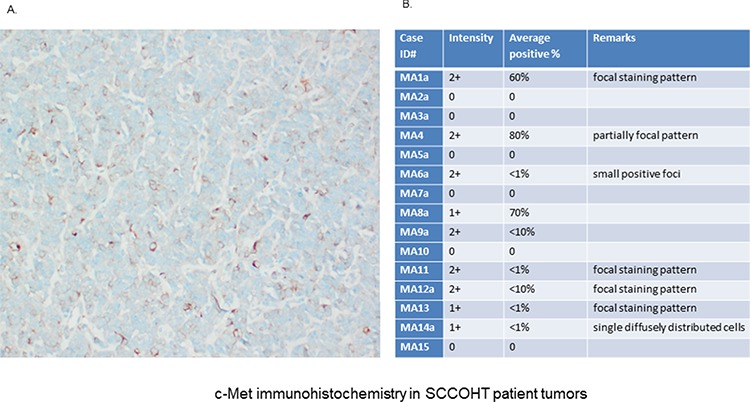
Immunhistochemical (IHC) detection of c-Met was performed in specimen of 16 different patients with confirmed SCCOHT **A.** IHC analysis in the primary patient tumor (origin of SCCOHT-1 cells) confirmed c-Met expression at variable levels with predominantly membrane-bound staining. **B.** An additional pilot series (*n* = 15) of primary SCCOHT showed microscopically detectable c-Met expression together with the SCCOHT-1 originating tumor predominantly in 6/16 cases. The staining confirmed significant c-Met expression in a subset of SCCOHT and showed striking variability across cases and within individual tumors, suggesting intra- and intertumoral heterogeneity of c-Met expression.

C-Met expression was also examined by immunohistochemistry (IHC) in 16 different SCCOHT patient tumor tissues (Fig. 3). The SCCOHT-1 cell-originating patient tumor demonstrated c-Met expression in about 40% of the cells (Fig. 3A) which is in line with our flow cytometry data. In addition, a series of 15 centrally reviewed primary SCCOHT was retrieved from the archives of the Mannheim referral center for gynecopathology by one of the authors (FK) and stained for c-Met expression by standard immunohistochemistry. While 6/15 tumors were c-Met negative, 4/15 tumors showed very low levels of c-Met expression (<1% positive cells), and 5/15 tumors were weakly to moderately c-Met positive (<10–80% positive cells, Fig. 3B). These results suggest heterogeneity of c-Met expression in individual SCCOHT tumors.

**Figure 4:**
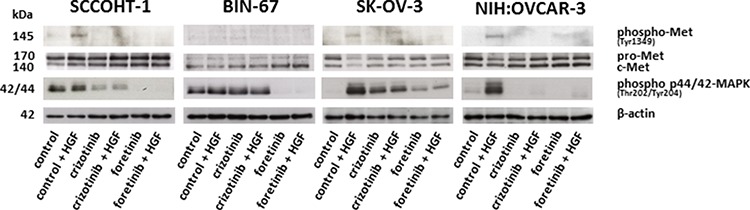
Western blot analysis was performed for c-Met phosphorylation at Tyr1349 and p44/p42 MAP kinase phosphorylation at Thre202/Tyr204 in SCCOHT-1, BIN-67, NIH:OVCAR-3 and SK-OV-3 ovarian cancer cells following 30 min of HGF stimulation in the presence or absence of the c-Met inhibitors crizotinib or foretinib, respectively

Accordingly, c-Met signaling was investigated in the different ovarian cancer cells. Exogenous stimulation of SCCOHT-1, SK-OV-3 and NIH:OVCAR-3 cells with 20 ng/ml HGF was associated with enhanced phosphorylation of c-Met at Tyr1349 after 30 min which was abolished in the presence of 2.5 μM crizotinib and 1.25 μM foretinib, respectively (Fig. 4). BIN-67 cells demonstrated barely detectable phosphorylation signals due to the low constitutive expression levels of c-Met (Fig. 4). Whereby constitutive c-Met expression remained unaltered, HGF stimulation was also accompanied by downstream signaling of enhanced p44/p42 MAP kinase phosphorylation at Thr202/Tyr204 particularly in SK-OV-3 and NIH:OVCAR-3 cells. Likewise, these HGF-mediated phosphorylation signals were partially diminished by a parallel incubation with 2.5 μM crizotinib and even more reduced in the presence of 1.25 μM foretinib (Fig. 4) suggesting the c-Met inhibitor foretinib a potent signaling inhibitor in these tumor cells.

**Figure 5:**
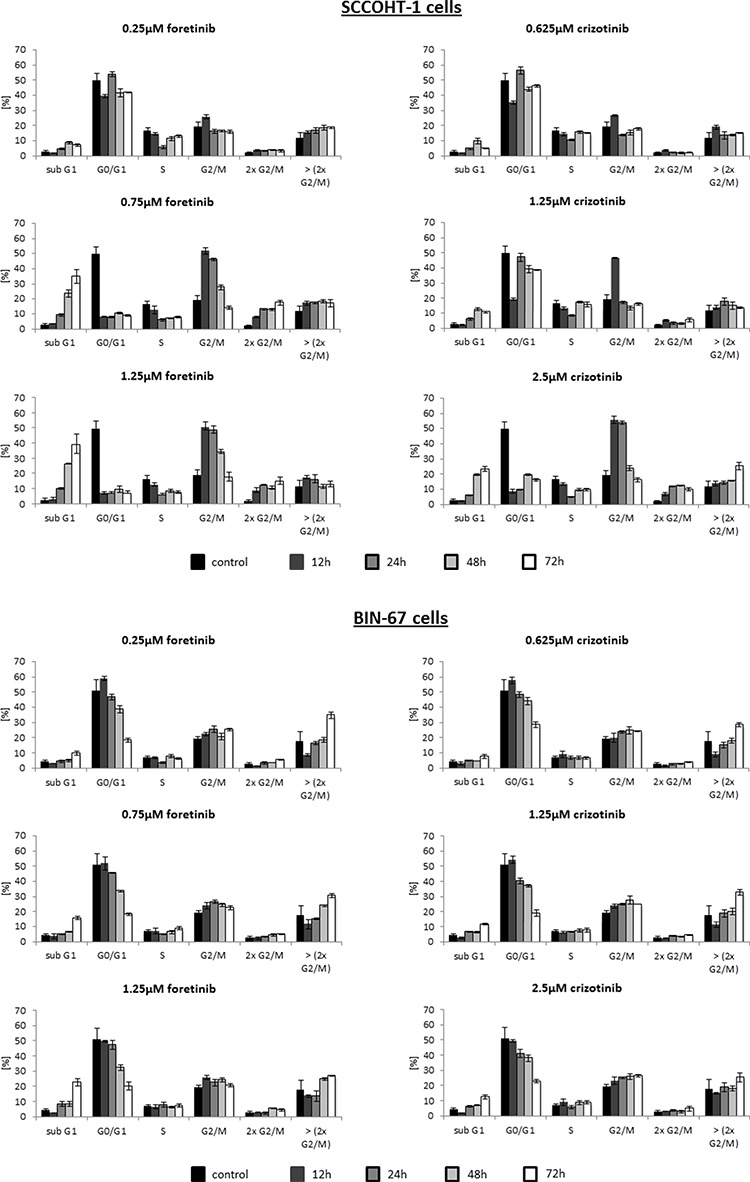

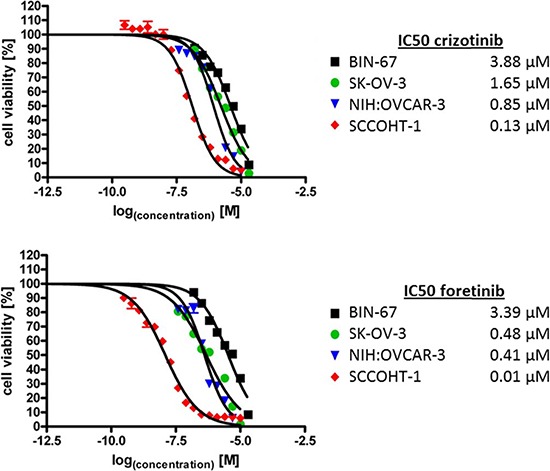
**A.** Cell cycle analysis was performed in SCCOHT-1, BIN-67 cells in the absence or presence of different concentrations of the c-Met inhibitors crizotinib and foretinib for 12 h up to 72 h, respectively. Quantification of the different cell cycle phases represent the mean ± s.d. (*n* = 3). **B.** SCCOHT-1^GFP^, BIN-67^GFP^, NIH:OVCAR-3^GFP^ and SK-OV-3^GFP^ ovarian cancer cells were incubated with different concentrations of crizotinib and foretinib for 72 h, respectively, and the proliferative capacity was measured by the fluoroscan assay. Analysis of a drug-dose-response to define IC50 concentrations for crizotinib and foretinib was performed using GraphPad Prism-6. For calculation of the drug-dose-response curves, the data were normalized to the cells-only control in culture medium and to the maximal solvent (DMSO) concentration control of the two compounds, respectively.

Effects of crizotinib and foretinib were also tested on the cell cycle progression and proliferative capacity of the ovarian cancer cells. Cell cycle analysis after incubation of SCCOHT-1 (Fig. 5A) and BIN-67 cells (Fig. 5B) with 0.25 μM up to 1.25 μM foretinib or 0.625 μM up to 2.5 μM crizotinib revealed a significant initial G2/M accumulation after 12 h which continuously declined until 72 h (Fig. 5A, 5B). This was paralleled by a progressively increasing apoptosis in sub G1 phase within 12 h to 72 h. Moreover, some aberrant mitosis was detectable by DNA doubling without cell division and therefore, appearance of populations with 2 × G2/M or more DNA content (>2 × G2/M) (Fig. 5A, 5B). A detailed time course of incubation with these c-Met inhibitors demonstrated similar effects also in NIH:OVCAR-3 and SK-OV-3 cells which substantiated a continuously increasing and significant G2/M phase accumulation already within 8 h to 12 h in the 4 different cell types (Suppl. Figs. S2A to S2D).

Examination of the IC_50_ for the two c-Met inhibitors demonstrated an about 10-fold increased sensitivity of SCCOHT-1 cells for foretinib (12.4 nM) compared to crizotinib (128 nM). Moreover, the ovarian adenocarcinoma cells NIH:OVCAR-3 and SK-OV-3 exhibited an about 2- and 3-fold enhanced sensitivity for foretinib whereas only little and insensitive differences were observed for BIN-67 cells (Fig. 5C). Together, these findings suggested a more specific growth inhibition associated with foretinib.

**Figure 6:**
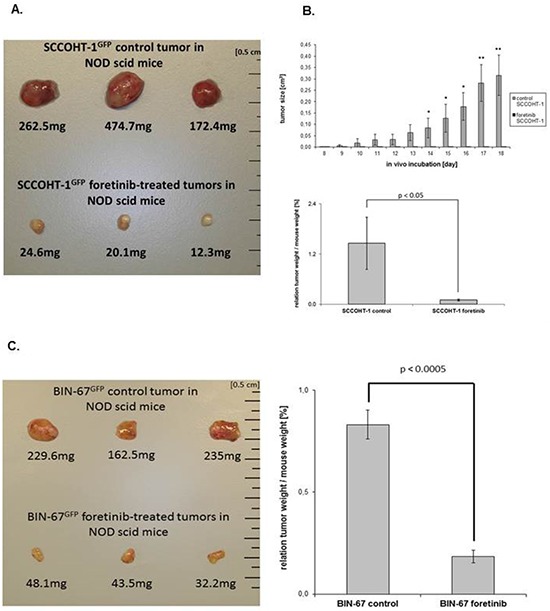

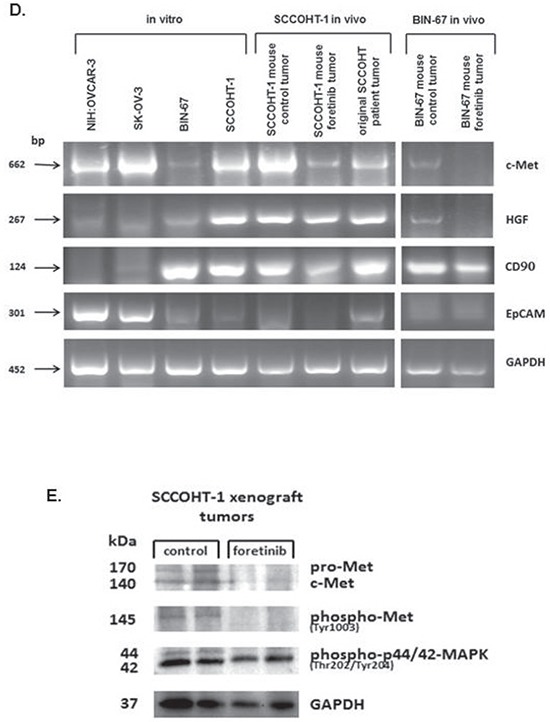

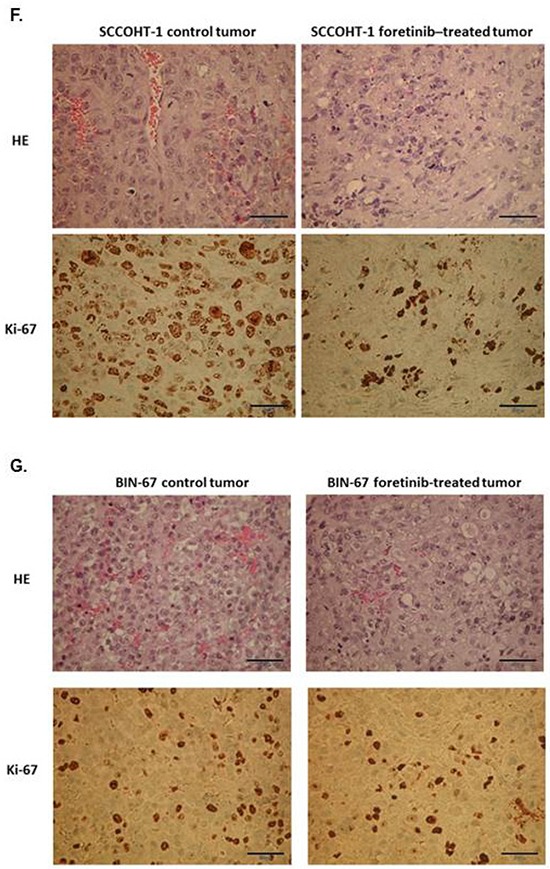
A. Size and weight of SCCOHT-1^GFP^-induced tumors in NOD^scid^ mice was compared in the absence or presence of a 10 days therapeutic approach with foretinib Following initial tumor detection, daily oral application was performed in 3 mice with 200 μl foretinib at a concentration of 50 mg/kg dissolved in 30% (v/v) propylene glycol, 5% (v/v) Tween 80, and 65% (v/v) of a 5% (w/v) dextrose solution in H_2_O. The other 3 mice were used as controls by a daily oral application of 200 μl of the solvent (30% (v/v) propylene glycol, 5% (v/v) Tween 80, and 65% (v/v) of a 5% (w/v) dextrose solution in H2O). After 10d of therapy, all 6 mice were sacrificed by cervical dislocation and the GFP-positive tumors were dissected under UV light, washed in PBS, and weighted. **B.** The tumor size (upper panel) of SCCOHT-1^GFP^-induced tumors in NOD^scid^ mice in the absence or presence of foretinib treatment was evaluated each day at 10 consecutive days of daily oral application as the mean ± s.d. for control tumors (*n* = 3) and foretinib-treated tumors (*n* = 3). Statistical analysis was calculated by unpaired Student's *t*-test (*P < 0.05; **P < 0.01). In the bottom panel, the relation of SCCOHT-1 tumor weight / mouse weight was calculated after 10d of subsequent treatment as the mean ± s.d. for control tumors (*n* = 3) and foretinib-treated tumors (*n* = 3). Statistical analysis was conducted by unpaired Student's *t*-test (*P < 0.05). **C.** Size and weight of BIN-67^GFP^-induced tumors in NOD^scid^ mice was compared in the absence or presence of a 10 days therapeutic approach with foretinib (left panel). The relation of BIN-67 tumor weight / mouse weight (right panel) was calculated after 10d of subsequent treatment as the mean ± s.d. for control tumors (*n* = 3) and foretinib-treated tumors (*n* = 3). Statistical analysis was conducted by unpaired Student's *t*-test (*P < 0.0005). **D.** The mRNA expression levels of various genes were analyzed by RT-PCR in the 4 ovarian cancer cell lines *in vitro* and compared to the *in vivo* NOD^scid^ SCCOHT-1 xenograft tumors together with the original patient tumor and to the *in vivo* BIN-67^GFP^ xenograft tumors following daily oral foretinib application for 10 consecutive days. **E.** Analysis of c-Met protein expression and associated phosphorylation signals in 2 control tumor xenografts (#1.2 and #1.3) was compared to 2 foretinib-treated tumor xenografts (#2.3 and #2.4) by Western blot analysis. **F.** Tissue sections (4 μm) were prepared by hematoxylin/eosin staining (HE) of SCCOHT-1^GFP^-induced control and foretinib-treated tumors in NOD^scid^ mice (upper panel; bars represent 50 μm). In addition, tissue sections (4 μm) of control and foretinib-treated tumor xenografts were compared by immune histochemistry using the proliferation marker Ki-67 (lower panel; bars represent 50 μm). **G.** Tissue sections (4 μm) were prepared by hematoxylin/eosin staining (HE) of BIN-67^GFP^-induced control and foretinib-treated tumors in NOD^scid^ mice (upper panel; bars represent 50 μm). In addition, tissue sections (4 μm) of control and foretinib-treated tumor xenografts were compared by immune histochemistry using the proliferation marker Ki-67 (lower panel; bars represent 50 μm).

To test potential growth-inhibitory effects of foretinib *in vivo*, xenograft tumors were induced in NOD^scid^ mice by the two SCCOHT cell lines. Following a subcutaneous injection of 3 × 10^6^ cells SCCOHT-1-induced mouse tumors (*n* = 3) could be detected already after 8d. A daily oral application of 200 μl foretinib (50 mg/kg) for 10 subsequent days revealed a white/yellow-colored tumor tissue with an about 10- to 20-fold reduced tumor mass compared to red-colored SCCOHT-1 control tumors (*n* = 3) after similar daily treatment with the solvent only (Fig. 6A). Following continuous tumor size measurements with corresponding calculation of the tumor volume, progressively increasing control tumors volumes were observed in contrast to an unaltered size of foretinib-treated tumors (Fig. 6B, upper panel). The relation of tumor weight /mouse weight after 10d of treatment revealed 1.46 ± 0.62% (*n* = 3) in control tumors and an about 15-fold reduced relation of 0.10 ± 0.02% (*n* = 3) in foretinib-treated SCCOHT-1 tumors (Fig. 6B, lower panel). Effects of foretinib treatment on the body weight of mice carrying SCCOHT-1 tumor xenografts revealed less than 20% differences (up to 17%; *n* = 3; *p* < 0.01) compared to control treatment after 10d (Suppl. Fig. S4, left panel).

BIN-67-induced mouse tumors (*n* = 3) appeared after 71d of subcutaneous tumor cell injection which took much longer to develop than SCCOHT-1 xenografts. This is also supported by the significantly reduced proliferative capacity of BIN-67 cells with an average cell doubling of approximately 75 h to 90 h compared to SCCOHT-1 cells representing a cell doubling time between 24 h to 36 h (Suppl. Fig. S3).

A similar foretinib treatment of BIN-67-induced mouse tumors (daily oral application of 200 μl foretinib (50 mg/kg) for 10d) was associated with an approximately 5-fold reduced tumor mass (Fig. 6C, left panel) and accordingly, the relation of tumor weight / mouse weight after 10d of treatment declined by 4.6-fold from 0.83 ± 0.07% (*n* = 3) in control tumors to 0.18 ± 0.03% (*n* = 3) in foretinib-treated BIN-67 tumors (Fig. 6C, right panel). In parallel, the body weight of mice carrying BIN-67 tumor xenografts remained relatively constant and revealed no significant differences during foretinib treatment (Suppl. Fig. S4, right panel).

Further analysis by RT-PCR revealed a strong c-Met expression in SK-OV-3 and NIH:OVCAR-3 cells and low levels in BIN-67 cells. Expression of c-Met in SCCOHT-1 cells was also detectable in the original SCCOHT patient tumor and in SCCOHT-1-induced and BIN-67-induced tumor xenografts whereas a reduced expression appeared in both foretinib-treated tumors (Fig. 6D). Low levels of HGF transcripts in NIH:OVCAR-3, SK-OV-3, and BIN-67 cells were paralleled by nearly unaltered levels in SCCOHT-1 cells and the SCCOHT-1 *in vivo* tumors whereas the low HGF expression in BIN-67 *in vivo* tumors decreased to undetectable signals after foretinib treatment. Differences in the CD90 and EpCAM mRNA transcripts between the ovarian adenocarcinoma NIH:OVCAR-3 and SK-OV-3 compared to BIN-67 and SCCOHT-1 cells and associated *in vivo* tumors substantiated the special entity of SCCOHT tumors (Fig. 6D).

Analysis of c-Met protein expression in control tumor xenografts by Western blot revealed a reduction in both, the 170kDa preform and the 140kDa active HGF receptor following a 10 day foretinib treatment of the mice. Moreover, the phosphorylation signal of c-Met at Tyr1003 present in the control tumors was abolished after foretinib application (Fig. 6E). In addition, downstream signaling by phosphorylation of p44/42 MAPK remained undetectable in foretinib-treated tumors whereby GAPDH expression was used as a control (Fig. 6E). These data suggested that the significantly reduced tumor size was associated at least in part with interruption of c-Met signaling followed by growth arrest after foretinib exposure. Supportive data were obtained by immunohistochemistry of the *in vivo* SCCOHT-1 (Fig. 6F) and BIN-67 (Fig. 6G) tumors. Staining with hematoxylin/eosin (HE) revealed various mitotic tumor cells in control tumor tissue in the vicinity of capillaries and microvessels. In contrast, less mitotic tumor cells and a significantly reduced vascularization were observed in the foretinib-treated tumors (Fig. 6F and 6G, upper panel). Moreover, the proliferation marker Ki-67 stained the majority of cells in the control tumor (about 91% in SCCOHT-1 and 31% in BIN-67) whereas only about 54% of Ki-67-positive SCCOHT-1 cells and about 17% of Ki-67-positive BIN-67 cells were detectable after foretinib application (Fig. 6F and 6G, lower panel). Together, these findings substantiated an attenuation of SCCOHT-1- and BIN-67-mediated tumor growth paralleled by a reduced vascularization in the yellow foretinib-treated tumors, respectively.

**Figure 7:**
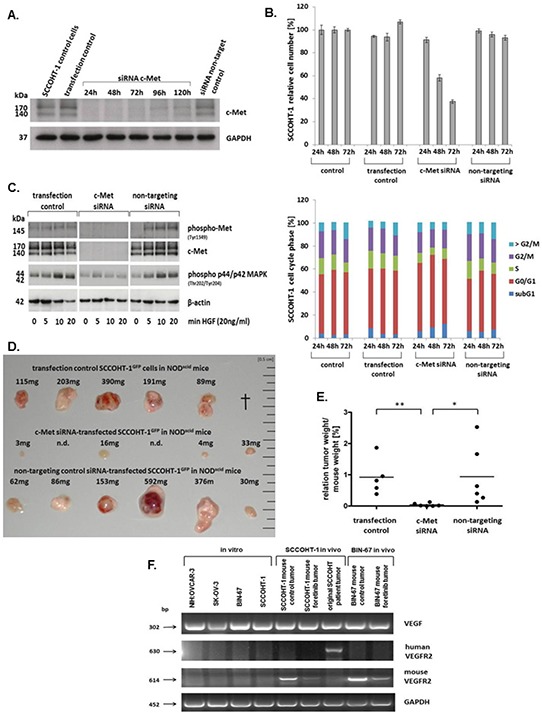
A. Western blot of c-Met in SCCOHT-1 steady state control cells, in cells using the transfection reagent alone (transfection control), and in cells with 25 nM of a non-targeting control siRNA was performed 24 h after transfection For c-Met siRNA-transfected SCCOHT-1 cells Western blots were performed between 24 h and 120 h post transfection. Analysis for GAPDH served as a loading control. **B.** Proliferative capacity (upper panel) and percentage of cell cycle phases (lower panel) of steady state SCCOHT-1 cells (control) was compared to cells after 24 h in the presence of the transfection reagent (transfection control), to cells transfected with c-Met siRNA (c-Met siRNA) and to cells transfected with a non-targeting siRNA (non-targeting siRNA). Cell numbers were counted for 24 h to 72 h and normalized ot the cell number of control cells (=100%). Data represent the mean ± s.d. of 3 independent experiments. **C.** Western blot of c-Met signaling was examined in SCCOHT-1 cells incubated with the transfection reagent for 24 h (transfection control), in cells transfected with 25 nM c-Met siRNA (c-Met siRNA), and in cells transfected with 25 nM of a non-targeting siRNA (non-targeting siRNA). In comparison to non-stimulated cells (0), the different populations were incubated with 20 ng/ml HGF for 5 min, 10 min, and 20 min, respectively. Expression of β-actin was used as a loading control. **D.** *In vivo* tumor development was evaluated after 22d of subcutaneous injection of 3 × 106 SCCOHT-1^GFP^ cells 24 h after transfection into 6 NOD^scid^ mice, respectively. Steady state control cells with transfection reagent (upper row) were compared to c-Met siRNA-mediated tumors (middle row) and tumors of a non-targeting control siRNA (lower row). In the control tumor section 1 mouse died in the course of the experiment (†, upper row). In the c-Met siRNA-transfected SCCOHT-1 cells 2 tumor developments were not detectable (n.d., middle row). Each tumor was verified by fluorescence microcopy demonstrating GFP expression. **E.** The relation of tumor weight / mouse weight of tumors from Fig. 7D was calculated Statistical analysis was conducted by unpaired Student's *t*-test (*P < 0.05; **P < 0.005). **F.** The mRNA expression levels of VEGF and human and mouse VEGFR2 was analyzed by RT-PCR in the 4 ovarian cancer cell lines *in vitro* and compared to the *in vivo* NOD^scid^ SCCOHT-1 and BIN-67^GFP^ xenograft tumors together with the original patient tumor of SCCOHT-1 cells. GAPDH expression levels served as a control.

The involvement of c-Met on cell growth was also tested by c-Met siRNA knock-down in SCCOHT-1. Transfection efficiency using an appropriate green fluorescing probe revealed about 92% (Suppl. Fig. S5). Effects of the c-Met siRNA were confirmed by Western blot analysis. A pronounced c-Met expression was detectable in control SCCOHT-1 cells, in cells using the transfection reagent alone (transfection control), and in cells transfected with 25 nM of a non-targeting control siRNA after 24 h (Fig. 7A). In contrast, down-modulation of c-Met protein was observed in c-Met siRNA-transfected SCCOHT-1 cells for up to 120 h (Fig. 7A). This down-modulation of c-Met in SCCOHT-1 caused a progressive growth reduction with a proliferative capacity of 35.1% ± 1.4% in c-Met siRNA-transfected cells as compared to about 100% in control cells and control transfectants after 72 h (Fig. 7B, upper panel). Moreover, c-Met siRNA-mediated growth inhibition in SCCOHT-1 cells was also accompanied by an accumulation of apoptotic cells in subG1 and an increase in G0/G1 cell cycle phase with reduced S phase (Fig. 7B, lower panel).

C-Met siRNA knock-down also abolished HGF-mediated phosphorylation signaling of the receptor. Whereas SCCOHT-1 transfection control and cells transfected with a non-targeting siRNA demonstrated increased c-Met phosphorylation at Tyr1349 as well as more downstream an increased p44/p42 MAP kinase phosphorylation at Thr202/Tyr204 after 5 min to 20 min of stimulation with 20 ng/ml HGF, little if any phosphorylation signal was observed in c-Met siRNA-transfected SCCOHT-1 cells (Fig. 7C). Likewise, expression of the c-Met receptor itself remained undetectable in c-Met siRNA-transfected SCCOHT-1 cells in contrast to constitutive c-Met expression in SCCOHT-1 transfection control and cells transfected with a non-targeting siRNA (Fig. 7C).

At a more functional level, down-modulation of c-Met in SCCOHT-1 cells also reduced tumor growth *in vivo*. Whereas 1 mouse with a control tumor died during the experiment, 5 mice developed pronounced and partially red-colored tumors within 22d (Fig. 7D, upper panel) indicating an appropriate tumor vascularization. Similar data were obtained from tumors of 6 mice injected with non-coding siRNA-transfected SCCOHT-1^GFP^ cells (Fig. 7D, lower panel). In contrast, mouse tumor xenografts of c-Met siRNA-transfected SCCOHT-1^GFP^ cells were observed only in 4/6 mice and appeared much smaller and less vascularized (Fig. 7D, middle panel). Calculation of the relation of tumor weight/mouse weight revealed 0.92 ± 0.51 (*n* = 5) for the transfection control and 0.94 ± 0.8 (*n* = 6) for the non-targeting siRNA-induced tumors. In contrast, c-Met siRNA-mediated tumors displayed an approximately 20-fold reduced ratio of 0.04 ± 0.04 (*n* = 6) (Fig. 7E).

Together, these data demonstrated an attenuation of tumor growth in SCCOHT-1 cells by targeting c-Met via antisense or via c-Met signaling interference compounds including foretinib. However, BIN-67 cells express only background levels of c-Met although foretinib treatment exhibited a significant tumor reduction which suggested additional effects of this compound. Indeed, foretinib treatment was also associated with interference of vascular endothelial growth factor (VEGF) signaling. Whereas VEGF was expressed by the ovarian tumor cell lines and by the SCCOHT tumors, transcripts of the related receptor VEGFR2 appeared species-specific and were observed exclusively in the corresponding human and mouse tumors, respectively (Fig. 7F). In particular, VEGFR2 expression exclusively in the original SCCOHT patient tumor but not in the corresponding SCCOHT-1 cells which were derived from this human tumor suggested paracrine VEGF effects by expression of this receptor in tumor-associated tissue rather than in the tumor cells. Moreover, foretinib treatment significantly reduced the VEGFR2 expression in the mouse tumors furthermore supporting additional effects of this drug also on the tumor microenvironment e.g. by reduced vascularization (Fig. 7F).

## DISCUSSION

SCCOHT represents a rare and aggressive tumor type with unclear etiology and insufficient therapeutic strategies. Whereas mutations in the *SMARCA4* gene and certain similarities to malignant rhabdoid tumors are known for this cancerous disease [14–16, 20] further characterization of the corresponding cellular models SCCOHT-1 and BIN-67 demonstrated significant differences in surface marker and filament expression compared to ovarian adenocarcinoma cells and therefore confirmed SCCOHT as a separate tumor entity with distinct growth properties. However, the growth factor receptor c-Met revealed common presences in the ovarian adenocarcinoma cells and to a lesser extend in SCCOHT-1 and BIN-67 cells.

The membrane receptor c-Met (Mesenchymal epithelial transition factor), also known as hepatocyte growth factor/scatter factor receptor is essential for embryonic development and wound healing [27]. In tumors, however, including ovarian cancer, c-MET overexpression and paralleled hyperactivation correlates with poor prognosis by triggering tumor growth, metastasis and angiogenesis [28–30]. Constitutive c-Met expression and enhanced angiogenic properties in SCCOHT-1 cells are also supported by their capacity of to produce and release VEGF and VCAM-1 into the tumor microenvironment which are essential for tumor neo-vascularization. Likewise, IL-8 (CXCL8) is associated with angiogenesis besides an involvement in inflammatory processes [31]. Moreover, the distinct chemokine/growth factor production by SCCOHT-1 cells including a constitutive HGF production suggested an autocrine loop for c-Met-relayed proliferation signals which could serve as a more specific therapeutic target in these cells. Additional exogenous HGF stimulation resulted in c-Met phosphorylation and enhanced downstream signaling by elevated p44/42-MAP kinase activation in SCCOHT-1 and the ovarian adenocarcinoma cells. Indeed, several phosphorylation sites are identified in the cytoplasmic c-Met domain to confer Erk/MAP kinase activation for induced proliferation and cell cycle progression in a variety of different tumor types including ovarian cancer [32–34]. Targeted approaches to antagonize aberrant c-Met signaling include therapeutic intervention in 1) ligand/receptor interaction, 2) inhibition of the tyrosine kinase catalytic activity, and 3) blockade of activated receptor/effector interaction. Thus, crizotinib and foretinib represent multi-targeted tyrosine kinase inhibitors to block c-Met signaling whereby foretinib was even more efficient to completely abolish both, constitutive and HGF-induced c-Met and subsequent MAP kinase phosphorylation in the ovarian adenocarcinoma as well as in the small cell hypercalcemic ovarian cancer cells. Simultaneously, these c-Met inhibitors effectively inhibited proliferation of the tumor cells by a pronounced G2/M cell cycle arrest and confirmed previous findings in foretinib-treated SK-OV-3 cells [35]. Since foretinib appears as a more potent inhibitor than crizotinib also in patients with ROS1-rearranged non-small-cell lung carcinoma [36], further *in vivo* studies revealed anti-tumorigenic effects of foretinib in lung metastasis [37] and in patients with sonic hedgehog-driven medulloblastoma [38]. In the present study, we could demonstrate a substantial therapeutic effect of foretinib by attenuating growth of SCCOHT-1- and BIN-67-induced tumors although SCCOHT-1 xenograft tumors developed much more rapidly. Both populations provide a cellular model for the rare SCCOHT whereby detectable c-Met expression was observed preferably in SCCOHT-1 cells and in the majority of SCCOHT tumor samples. Consequently, c-Met and associated targets by foretinib treatment represent a potential therapeutic approach for this tumor entity. Moreover, foretinib displayed additional effects by down-modulation of the VEGFR2 within the SCCOHT tumor microenvironment and thereby reduced cancer growth also in BIN-67-induced tumors with little or barely detectable c-Met expression.

Together, interference with c-Met signaling *in vivo* significantly diminished the SCCOHT-1 and BIN-67 tumor sizes, respectively. This effect was paralleled by a marked reduction of growth-associated structures in the tumor microenvironment such as tumor vascularization. Supportive evidence for anti-tumor activity of foretinib by inhibition of c-Met and vascular endothelial growth factor receptor 2 was obtained in models of hepatocellular carcinoma [39], renal cell carcinoma [40], and gastric cancer [41] which also underscores the importance to simultaneously target the tumor microenvironment by blocking neo-vascularization. In this context, interaction of SCCOHT-1 tumor cells by a close vicinity to mesenchymal stroma/stem cells in the tumor microenvironment can transfer proteins, RNAs and further biological material thereby altering cellular functionality and contributing to increased tumor heterogeneity [42–46].

In conclusion, the data suggested that in all presently available models of the rare SCCOHT tumor entity the attenuation of *in vivo* SCCOHT-1 or BIN-67 tumor growth and vascularization blockage by foretinib provides a specific target to further support a combination with cytotoxic agents in a promising therapeutic approach.

## MATERIAL AND METHODS

### Cell culture

#### Culture of human SCCOHT cells

Cellular models of SCCOHT are represented by the two cell lines BIN-67 and SCCOHT-1. BIN-67 were kindly provided by Dr. Barbara Vanderhyden (University of Ottawa, Canada) and cultured with DMEM/F12 : DMEM medium (1:1) (Sigma Aldrich, St. Louis, MO) supplemented with 20% (v/v) fetal calf serum, 2 mM L-glutamine, 100 U/ml penicillin and 100 μg/ml streptomycin [21].

SCCOHT-1 cells were generated in our lab as a spontaneously growing primary culture derived from a tumor biopsy of a 31-year-old patient with recurrent SCCOHT [18]. The study has been approved by the Ethics Committee of Hannover Medical School, Project #3916 on June 15th, 2005 and informed written consent was obtained from the patient for the use of this material. The SCCOHT-1 cells were cultured in RPMI 1640 supplemented with 10% (v/v) fetal calf serum, 100 U/ml L-glutamine, 100 U/ml penicillin and 100 μg/ml streptomycin. Serum-free cultures of SCCOHT-1 were maintained in HybridoMed DIF 1000 medium (Biochrom, Berlin, Germany). The cell culture was performed at 37°C in a humidified atmosphere of 5% (v/v) CO_2_ and the culture medium was changed at intervals of 3 to 4 days. For subculture, the cells were centrifuged (320 g/6 min) and resuspended in the appropriate growth medium.

#### Human ovarian adenocarcinoma cell lines

Human NIH:OVCAR-3 ovarian cancer cells (ATCC^®^ #HTB-161™) were commercially obtained in passage 76 (P76) from the Institute for Applied Cell Culture (IAZ), Munich, Germany. The SK-OV-3 ovarian cancer cells (ATCC^®^ #HTB-77™) were commercially obtained in P25 from the ATCC, Manassas, VA, USA. These two cell lines were originally established from the malignant ascites of a patient with progressive adenocarcinoma of the ovary, respectively. Both cell types were cultivated at about 1,750 cells/cm^2^ in RPMI 1640 supplemented with 10% (v/v) fetal calf serum, 100 U/ml L-glutamine, 100 U/ml penicillin and 100 μg/ml streptomycin. Subculture was performed by trypsin/EDTA (Biochrom GmbH, Berlin, Germany) treatment for 5 min at 37°C.

#### Cell line authentication

All cells were tested for mycoplasma by the luminometric MycoAlert Plus mycoplasma detection kit (Lonza Inc., Rockland, ME, USA) according to the manufacturer's recommendations. Moreover, authentication of SCCOHT-1, BIN-67, NIH:OVCAR-3, and SK-OV-3 cell lines was performed by short tandem repeat (STR) fragment analysis using the GenomeLab human STR primer set (Beckman Coulter Inc., Fullerton, CA, USA). The fragment analysis for the NIH:OVCAR-3 and SK-OV-3 cell lines demonstrated a similar STR pattern according to the STR database provided by the Deutsche Sammlung von Mikroorganismen und Zellkulturen (DSMZ, Braunschweig, Germany).

Whereas no STR patterns are available for SCCOHT-1 and BIN-67 cells to date, repeated STR fragment analyses were performed for these two cell populations. Data from different SCCOHT-1 culture periods (*n* = 3) confirmed similar patterns and these STR pattern were presented in Suppl. Fig. S6.

### Cytokine production and release

Following culture of 2 × 10^5^/ml SCCOHT-1 cells in serum-free HybridoMed DIF 1000 medium, supernatants were collected after 48 h and 72 h, respectively. The conditioned medium was filtered in a 0,2 μm filter (Sartorius Stedim Biotech GmbH, Göttingen, Germany) to remove cells and debris, and aliquots were shockfrozen in liquid nitrogen and stored at −80°C until measurement. Aliquots of the supernatants were applied to a Quantibody^®^ array (RayBiotech Inc., Norcross, GA, USA / tebu-bio GmbH, Offenbach, Germany), which represents a quantitative array platform by using a multiplexed sandwich ELISA-based technology. This method allows to simultaneously and quantitatively determine the concentration of multiple cytokines. Membranes of the Quantibody Human Chemokine Array (RayBiotech Inc./tebu-bio GmbH) were incubated with the cell supernatants in comparison to control medium in quadruplicates and developed by chemiluminescence according to the manufacturer's instructions. Chemokine concentrations were measured (based on internal controls) in pg/ml + s.d. (*n* = 4) using appropriate manufacturer's software.

### Analysis of surface markers by flow cytometry

Continuously proliferating SCCOHT-1, BIN-67, NIH:OVCAR-3 and SK-OV-3 cells in logarithmic growth phase were harvested and analyzed for cell surface marker expression by flow cytometry. After blocking non-specific binding to Fc-receptors by incubation of 10^6^ cells with 2% FCS for 30 min at 4°C and washing with PBS-BSA, the cells were incubated with the following appropriately-labeled monoclonal anti-human antibodies, respectively: c-Met-FITC (clone eBioclone97, IgG1, eBioscience, Inc., San Diego, CA, USA); CD29-PE (clone MAR4, IgG1, BD Biosciences, Heidelberg, Germany); CD44-FITC (clone G44–26, IgG2b, BD Biosciences); CD90-PE (clone 5E10, IgG1, BioLegend Inc., San Diego, CA, USA); CD105-PE (clone 43A3, IgG1, BioLegend Inc.); CD326-PE (clone G9C4, IgG2b, BioLegend Inc.); vimentin-PE (clone VI-RE-1, IgG1, antibodies-online Inc., Atlanta, GA, USA); pan-cytokeratin-PE (clone C-11, IgG1, Acris Antibodies GmbH, Herford, Germany); Following antibody staining, all samples were washed twice with PBS-BSA. Moreover, SCCOHT-1, BIN-67, NIH:OVACAR-3 and SK-OV-3 cells were incubated with a primary monoclonal anti-human mesothelin antibody (clone K1, IgG1, abcam plc, Cambridge, UK), respectively, followed by two washes with PBS-BSA and subsequent addition of a secondary PE-labeled polyclonal rabbit anti-mouse antibody (DakoCytomation Inc., Glostrup, Denmark).

A parallel incubation of the cells with appropriately-labeled antibodies of the corresponding Ig subclass were used as controls. Flow cytometry analysis was performed in a Galaxy FACSan (Partec) using FloMax analysis software (Partec).

### Immunohistochemical c-Met analysis in SCCOHT patient tumors

Immunohistochemistry was performed on an automated staining instrument (Benchmark Ultra; Ventana, Tuscon, U.S.A) using the CC1 mild antigen retrieval procedure and a monoclonal anti-c-Met antibody (clone D1C2, rabbit, Cell Signaling Technology, Danvers, MA, USA), diluted at 1:100 in primary antibody diluent following the manufacturer's recommendations. Semiquantitative slide evaluation by two observers (RH/FF) included scoring of staining intensity (0, 1+, 2+, 3+) and estimation of percentage of positive cells in 10% increments.

### Immunoblot analysis

Cell lysates of SCCOHT-1, BIN-67, NIH:OVCAR-3 or SK-OV-3 cells were prepared in reswelling buffer containing 8 M urea (Carl Roth GmbH Co KG, Karlsruhe, Germany), 1% CHAPS (3-[(3-Cholamidopropyl)-dimethylammonio]-1-propanesulfonate) (Carl Roth GmbH Co KG), 0.5% (v/v) Pharmalyte 3–10 (GE Healthcare Europe GmbH, Freiburg, Germany), 0.002% (w/v) bromophenol blue (SERVA Electrophoresis GmbH, Heidelberg, Germany) and freshly prepared 0.4% (w/v) DTT (Dithiothreitol) (Carl Roth GmbH Co KG). Protein concentration of the cell lysates was adjusted using the colorimetric BCA-assay (Thermo Scientific, Schwerte, Germany). Aliquots of 40 μg protein were subjected to SDS-polyacrylamide gel electrophoresis and transferred to a Amersham™ Protran™-Supported 0.45 μm nitrocellulose membrane (GE Healthcare). The membranes were blocked with PBS containing 5% low fat milk and 0.05% Tween-20 (PBS/Tween). After washing four times with PBS/Tween, the membranes were incubated with the primary antibodies: monoclonal anti-c-Met (clone D1C2, rabbit, (dilution 1:1,000); Cell Signaling Technology, Danvers, MA, USA); monoclonal anti-phospho-Met (Tyr1349) (clone 130H2, rabbit, (dilution 1:1,000); Cell Signaling Technology); monoclonal anti-phospho-Met (Tyr1003) (clone 13D11, rabbit, (dilution 1:1000), Cell Signaling Technology); polyclonal anti-phospho p44/42 MAP kinase (Thr202/Tyr204) (rabbit, (dilution 1:1,000), Cell Signaling Technology); monoclonal anti-GAPDH (clone 6C5, mouse, (dilution 1:200), Santa Cruz Biotechnology Inc., Dallas, Texas, USA); monoclonal anti-β-actin (clone AC-15, mouse, (dilution 1:1,000), Sigma, St. Louis, Missouri, USA). Thereafter, the membranes were washed four times with PBS/Tween and incubated with the appropriate horseradish peroxidase-conjugated anti-mouse IgG (dilution 1:5,000) or anti-rabbit IgG (dilution 1:2,000) secondary antibody, respectively, (all from GE Healthcare, Freiburg, Germany) for 1 h/room temperature. The membranes were washed with PBS/Tween and visualized by autoradiography using SuperSignal West Pico Chemiluminescent Substrate (Thermo Scientific, Schwerte, Germany).

### Cell cycle analysis

Cell cycle analysis compared to untreated controls was performed in 5 × 10^5^ SCCOHT-1, BIN-67, NIH:OVCAR-3 or SK-OV-3 cells after culture either in the presence of 0.025% (v/v) DMSO (Sigma) as solvent, 2.5 μM crizotinib, or 1.25 μM foretinib (= GSK1363089; = PF-02341066) (both from Selleck Chemicals LLC, Houston, TX, USA) for up to 72 h, respectively. The cells were fixed in 70% (v/v) ice-cold ethanol at 4°C for 24 h. Thereafter, the fixed cells were stained with CyStain DNA 2 step kit (Partec GmbH, Münster, Germany) and filtered through a 50 μm filter. Flow cytometry analysis was performed in a Galaxy FACSan (Partec) using FloMax analysis software (Partec).

### Proliferation rate

The proliferative capacity and the sensitivity of SCCOHT-1, BIN-67, NIH:OVCAR-3 and SK-OV-3 cells was determined for different concentrations of crizotinib and foretinib. In a fluorescence-based proliferation assay the ovarian cancer cell types were transduced with a 3rd generation lentiviral SIN vector containing the eGFP gene as previously described for these cells [18, 19]. The different eGFP-transduced ovarian cancer cell populations (SCCOHT-1^GFP^, BIN-67^GFP^, NIH:OVCAR-3^GFP^ and SK-OV-3^GFP^) were incubated with culture medium in flat bottom 96-well plates (Nunc/ThermoFischer) at a density of 3 × 10^3^ cells/well and following incubation for 24 h, 48 h, and 72 h, the medium was removed and the cells were lysed with 5% (w/v) SDS. Afterwards, the fluorescence intensities of GFP in the cell homogenate which corresponded to the appropriate cell number of ovarian cancer cells was measured at excitation 485 nm / emission 520 nm using the Fluoroscan Ascent Fl (Thermo Fisher Scientific).

### *In vivo* experiments

Animal research using NOD^scid^ mice was carried out by following internationally recognized guidelines on animal welfare and has been approved by the institutional licensing committee ref. #33.14–42502-04–12/0814 on June 26th, 2012.

About 3 × 10^6^ SCCOHT-1^GFP^ or BIN-67^GFP^ cells were injected subcutaneously into 5 weeks old female NOD^scid^ mice (*n* = 6 for each cell line). Within 8 days, the 6 SCCOHT-1-treated mice and after 71d the other 6 BIN-67-treated mice had developed small subcutaneous tumors. Systemic therapy was performed in 3 mice of both cell line-induced tumors by a daily oral application of 200 μl foretinib (GSK1363089) (50 mg/kg) (Selleck Chemicals LLC) dissolved in 30% (v/v) propylene glycol, 5% (v/v) Tween 80, and 65% (v/v) of a 5% (w/v) dextrose solution in H_2_O. The other 3 mice of both cell line-induced tumors were used as controls by a daily oral application of 200 μl of the solvent (30% (v/v) propylene glycol, 5% (v/v) Tween 80, and 65% (v/v) of a 5% (w/v) dextrose solution in H_2_O). Although foretinib in clinical studies is administered to patients between 3.6 mg/kg to 4.5 mg/kg [47], *in vivo* mouse experiments including recent studies are performed at foretinib concentrations of 60 mg/kg to 100 mg/kg [38].

Oral application was performed using plastic feeding tubes (18 ga × 30 mm) (Instech Laboratories, Plymouth, PA, USA). Following 10d of therapy, the 6 mice of each cell line-induced tumors were sacrificed by cervical dislocation. Tumor volumes (V) of SCCOHT-1 tumors were calculated with the longitudinal diameter (length) and the transverse diameter (width) in the modified ellipsoidal formula V = π/6 * width * (length)^2^ [48].

The GFP-positive tumors were dissected under UV light, washed in PBS, weighted and either cryo-preserved in liquid nitrogen for subsequent PCR and Western blot analysis or fixed in 4% glutardialdehyde solution for histopathological evaluations.

### Transcript analysis by RT-PCR

Total RNA was isolated from cells and tumor tissues using RNeasy Mini Kit (Qiagen, Hilden, Germany) according to the manufacturer's instructions. One μg RNA was reverse transcribed into cDNA using 500 μM of dNTP (R0193), 5 μM Oligo(dT)18 primer (S0132), 5 μM Random Hexan primer (S0142), 1 U RiboLockTM RNase Inhibitor (E00381) and 5 U RevertAidTM M-MuLV Reverse Transcriptase (EP0441) in the supplied reaction buffer (all reagents from Thermo Scientific, Schwerte, Germany). The cDNA reactions were performed for 10 min/25°C, 1 h/37°C and stopped at 72°C for 10 min. As a template 2.5 μl of cDNA was used with primers specific for:

- Hepatocyte growth factor (scatter factor) (HGF) (sense: 5′-AGG AGA AGG CTA CAG GGG CAC-3′; antisense: 5′-TTT TTG CCA TTC CCA CGA TAA-3′; amplification product 267 bp) [49];
- c-Met (sense: 5′-CAG GCA GTG CAG CAT GTA GTG-3′; antisense: 5′-TAA GGT GGG GCT CCT CTT GTC A-3′; amplification product 662 bp) [50];
- CD90 (sense: 5′-GGA CTG AGA TCC CAG AAC CA-3′; antisense: 5′-ACG AAG GCT CTG GTC CAC TA-3′; amplification product 124 bp) [51]);
- EpCAM (sense: 5′-GAA GGC TGA GAT AAA GGA GAT GGG-3′; antisense: 5′-TTA ACG ATG GAG TCC AAG TTC TGG-3′ amplification product 301 bp) [52];
- VEGF-A (sense: 5′-CCT CAG TGG GCA CAC ACT CC-3′; antisense: 5′-CGA AAC CAT GAA CTT TCT GC-3′ amplification product 302bp) [53]
- human VEGF-R2 (sense: 5′-TTA CAG ATC TCC ATT TAT TGC-3′; antisense: 5′-TTC ATC TCA CTC CCA GAC T-3′ amplification product 630bp) [54]
- mouse VEGF-R2 (sense: 5′-ATA ACC TGG CTG ACC CGA TTC-3′; antisense: 5′-TCG GTG ATG TAC ACG ATG CC-3′ amplification product 614 bp)
- GAPDH as a control PCR (sense: 5′-ACC ACA GTC CAT GCC ATC AC-3′; antisense: 5′-TCC ACC ACC CTG TTG CTG TA-3′; amplification product 452 bp [55])

was performed (all primers customized by Eurofins, MWG GmbH, Ebersberg, Germany). PCR reactions included 0.2 μM of each primer, 200 μM of dNTP (R0193, Thermo Scientific) and 0.05 U Taq DNA Polymerase (EPO402, Thermo Scientific) in the supplied reaction buffer. PCR cycling conditions were performed 30 sec at 94°C, 1 min at 60°C and 72°C for 1 min respectively, including an initial 30 sec denaturation step at 94°C and a final 10 min extension step at 72°C (35 cycles). Aliquots of 25 μl of each RT-PCR product were separated on a 2% agarose gel including the standard GeneRuler 100 bp DNA Ladder (Thermo Scientific) and visualized by GelRedTM (Biotium Inc., Hayward, CA, US) staining.

### siRNA knock-down of c-Met

For c-Met knock-down a transfection protocol was applied according to the manufacturer's instructions (Dharmacon, GE Healthcare, Uppsala, Sweden) using c-Met small interfering RNA (siRNA). Briefly, SCCOHT-1 cells were transfected with 25 nM c-Met siRNA (siGENOME human MET SMARTpool, cat. #D-003156-02) or with 25 nM of a non-targeting control siRNA (non-targeting #3 control, cat. #D-001210-03, Dharmacon, GE Healthcare, Uppsala, Sweden) using a 1:1,000 dilution of the DharmaFECT 4 transfection reagent (Dharmacon) in transfection medium (RPMI-1640 medium supplemented with 2% (v/v) fetal calf serum, 100U/ml L-glutamine) for 24 h. For evaluation of the transfection efficiency, SCCOHT-1 cells were transfected with 25 nM of the green fluorescing siGLO^green^ (cat. #D-001630-01, Dharmacon). Thereafter, the cells were washed and cultured in normal growth medium.

## SUPPLEMENTARY FIGURES


