# Supplementary material for: c-Met inhibitors attenuate tumor growth of small cell hypercalcemic ovarian carcinoma (SCCOHT) populations
Source: Oncotarget. 2015 Sep 30;6(31):31640–58. doi: 10.18632/oncotarget.5151 (PMC4741630; doi:10.18632/oncotarget.5151)
Supplement: Supplementary file 1 [file oncotarget-06-31640-s001.pdf]

SUPPLEMENTARY FIGURES

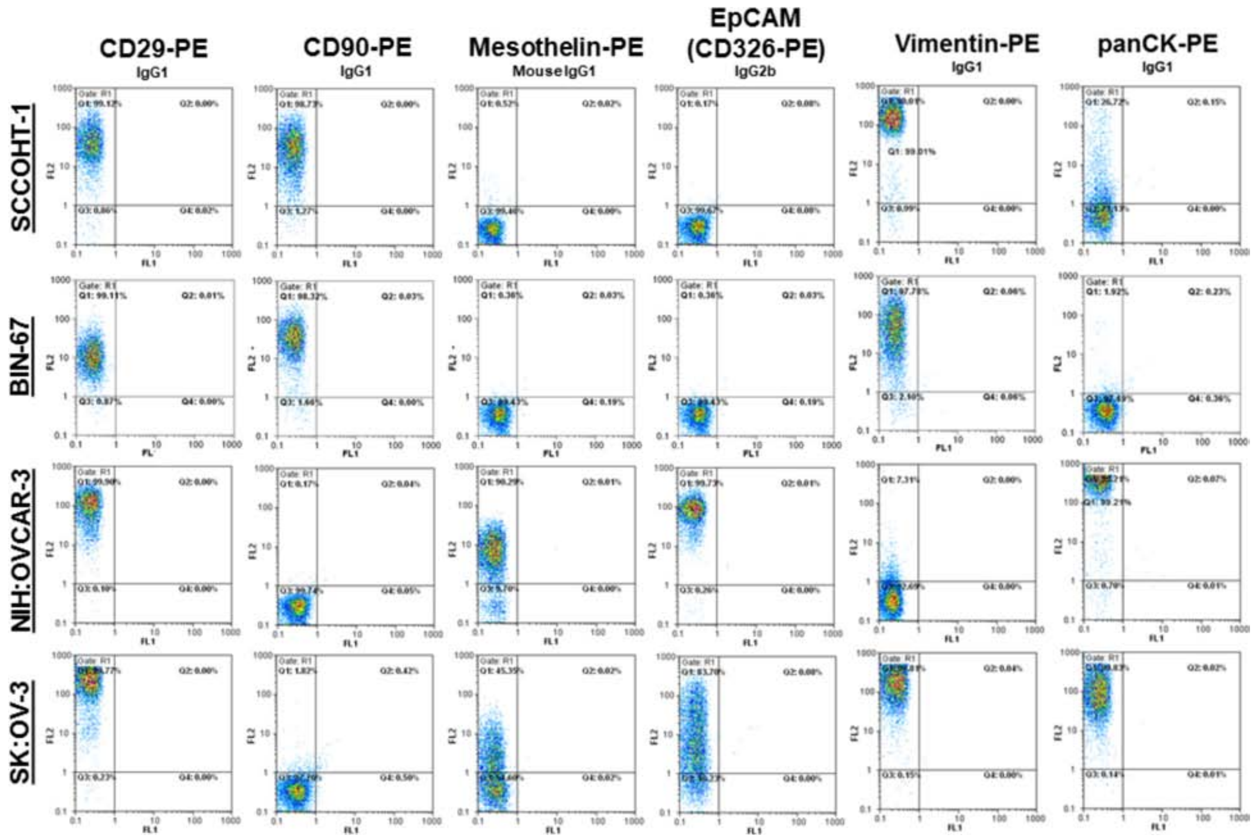

Supplementary Figure S1: Cell marker analysis was performed for a variety of different surface molecules and intermediate filament proteins (vimentin, cytokeratin (panCK)) in steady state-growing SCCOHT-1, BIN-67, NIH:OVCAR-3 and SK-OV-3 ovarian cancer cells by flow cytometry analysis according to the appropriate Ig isotype control.

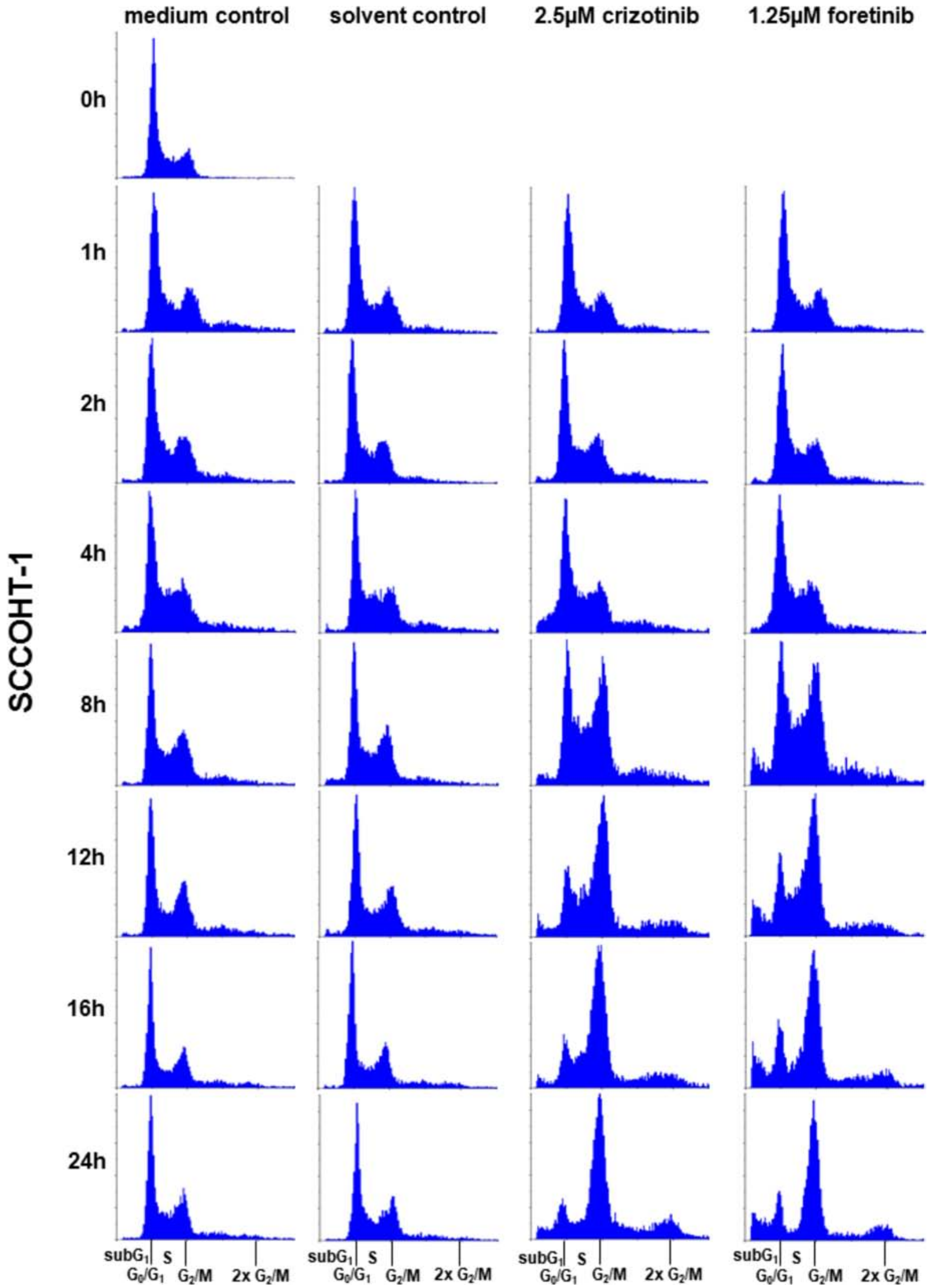
medium control
solvent control
2.5µM crizotinib
1.25µM foretinib
SCCOHT-1
0h
1h
2h
4h
8h
12h
16h
24h
subG1
S
G0/G1
G2/M
2x G2/M

(*Contiuned*)

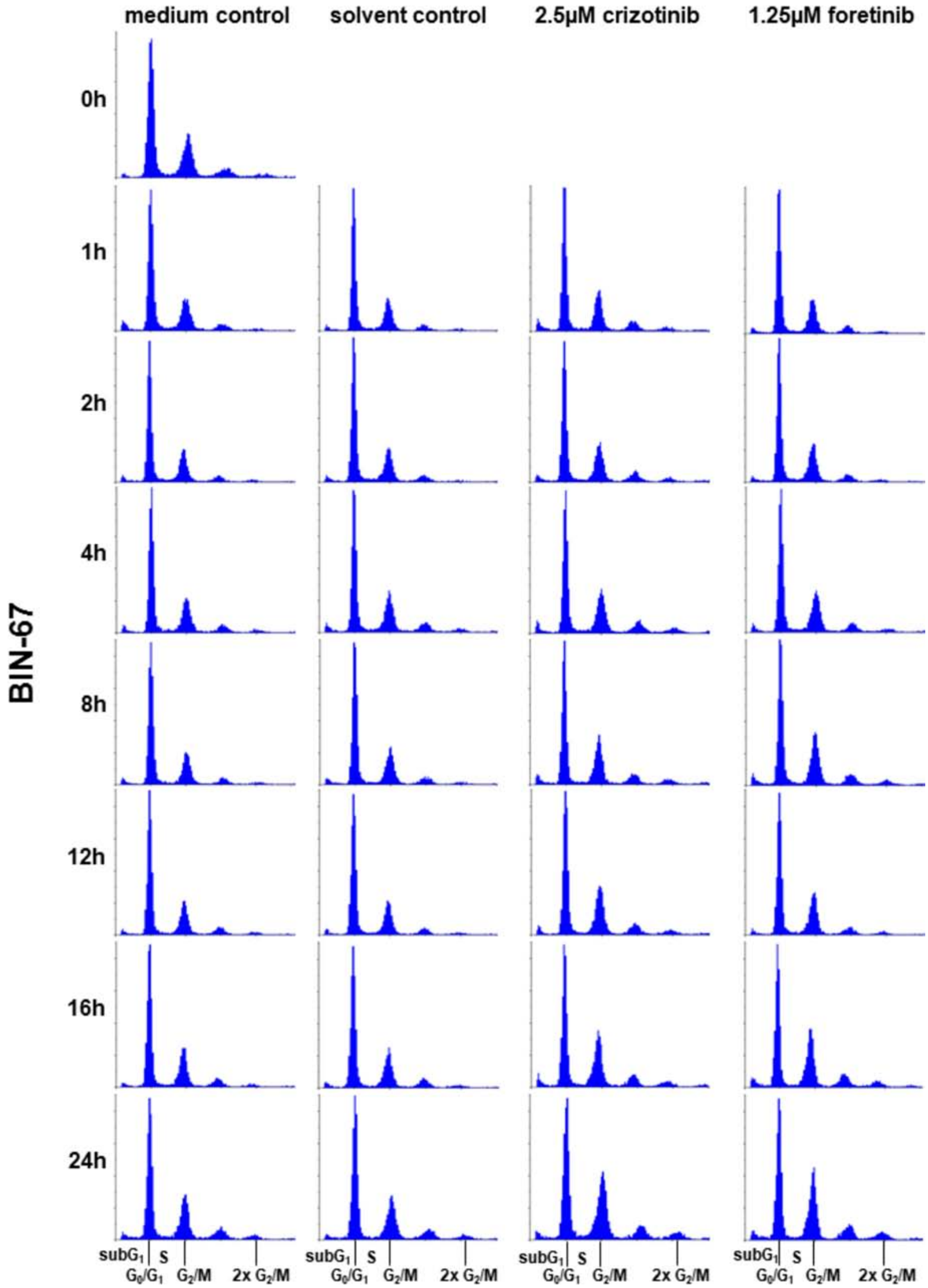
medium control
solvent control
2.5μM crizotinib
1.25μM foretinib
0h
1h
2h
4h
8h
12h
16h
24h
BIN-67
subG1
S
G0/G1
G2/M
2x G2/M

(*Contiuned*)

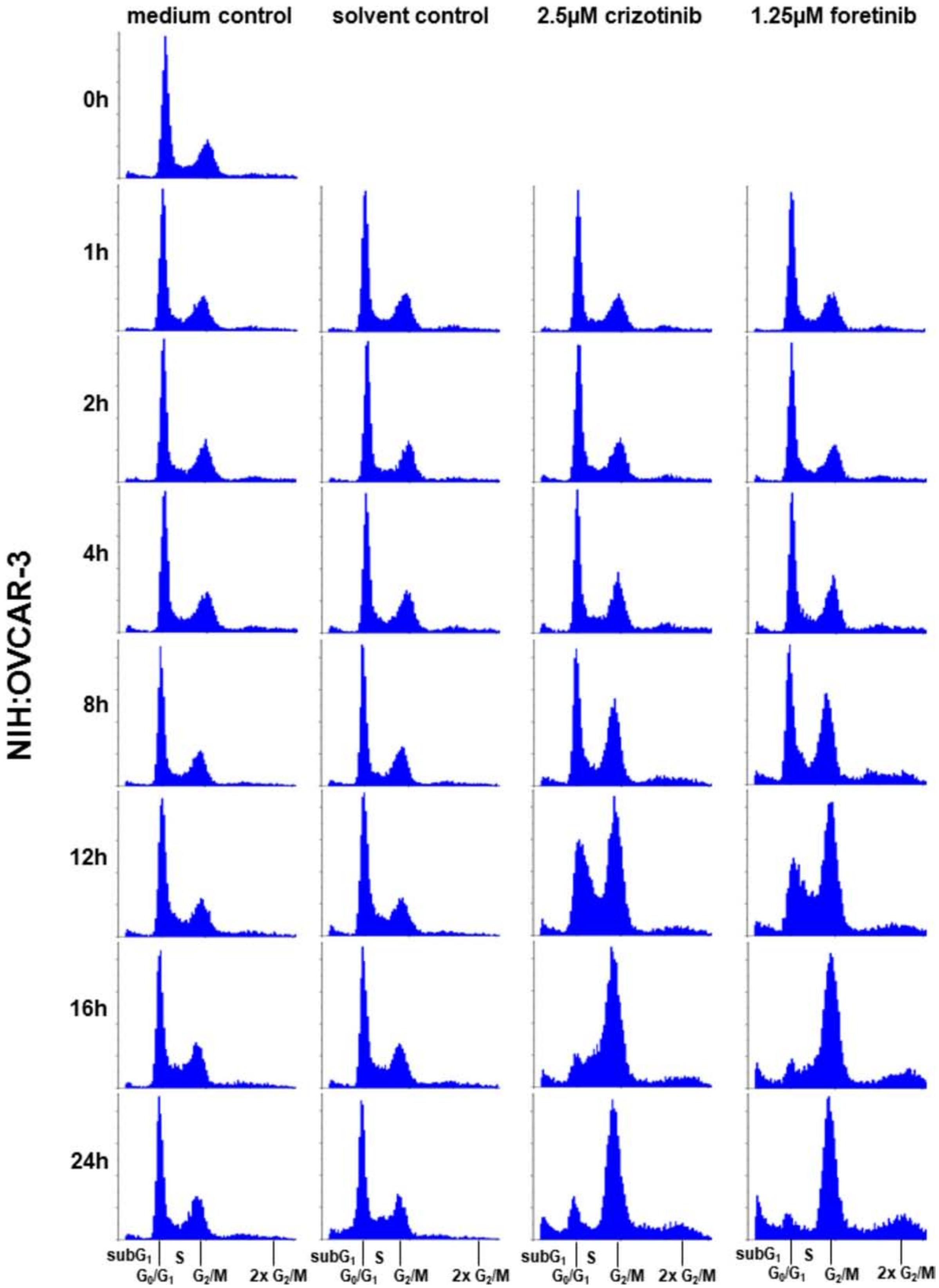
medium control
solvent control
2.5μM crizotinib
1.25μM foretinib
NIH:OVCAR-3
0h
1h
2h
4h
8h
12h
16h
24h
subG1
S
G0/G1
G2/M
2x G2/M

(*Contiuned*)

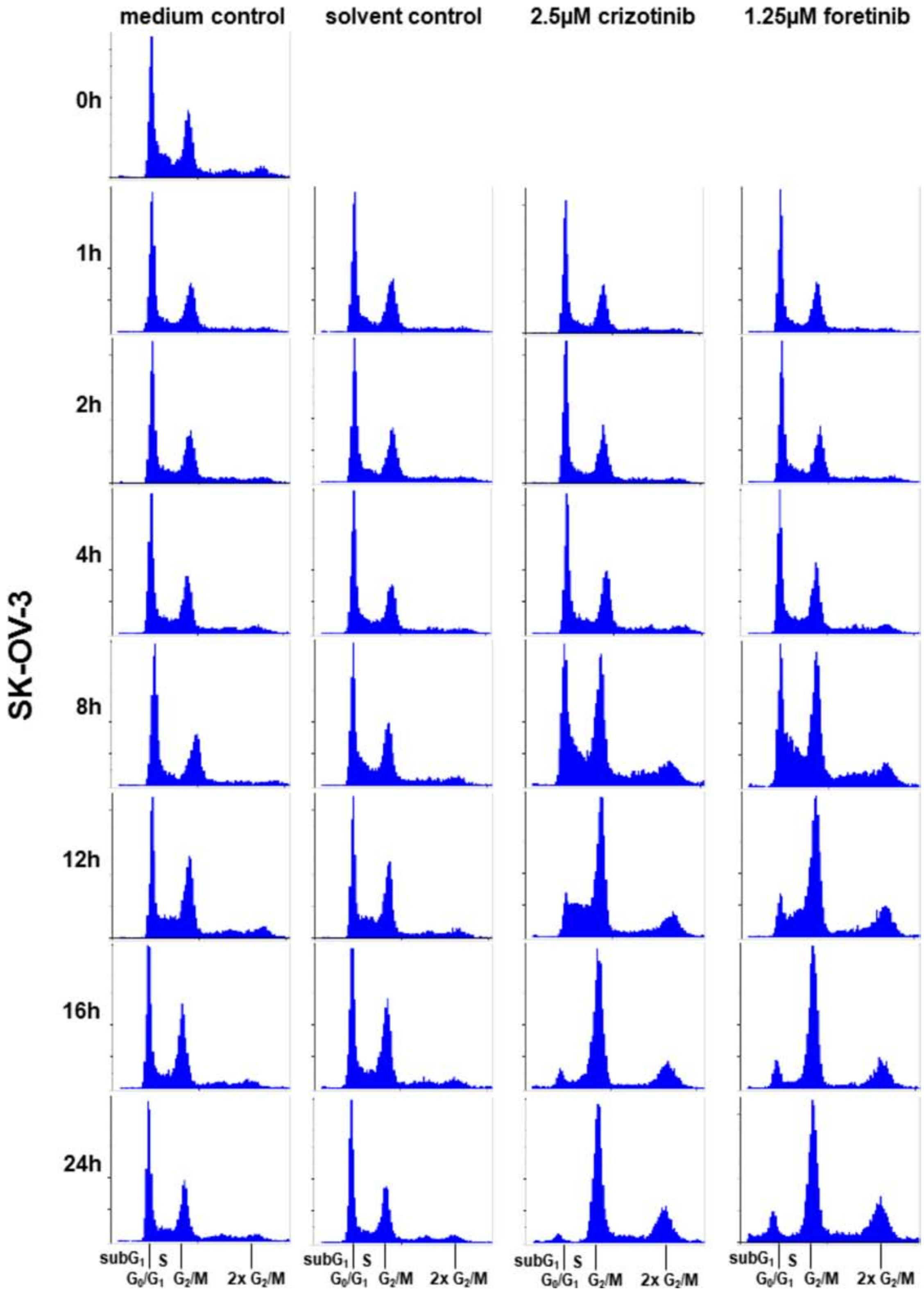

Supplementary Figure S2: Short term cell cycle analysis of A. SCCOHT-1, B. BIN-67, C. NIH:OVCAR-3 and D. SK-OV-3 ovarian cancer cells was performed in steady state controls, in solvent controls (0.025% (v/v) DMSO), or in the presence of the c-Met inhibitors 2.5 µM crizotinib and 1.25 µM foretinib for up to 24 h, respectively.

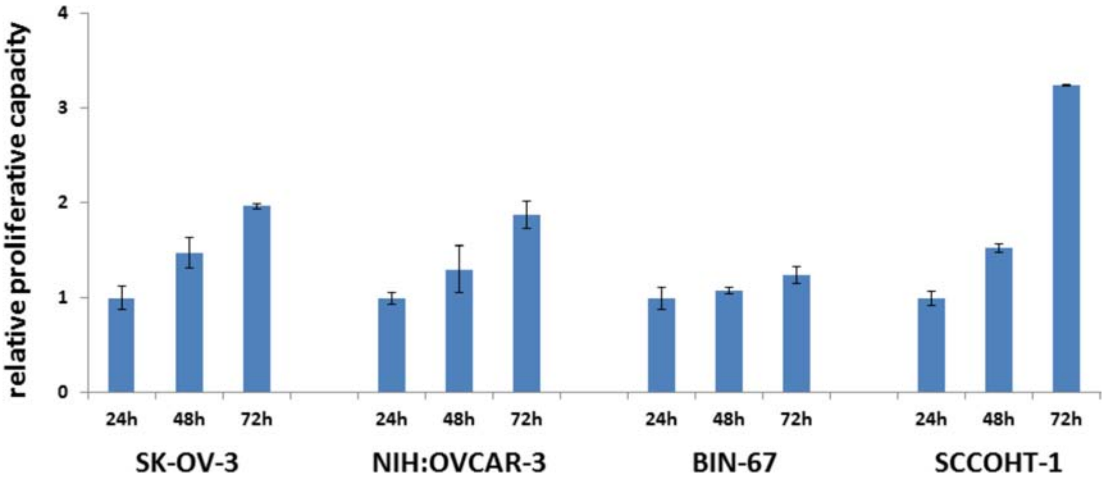

Supplementary Figure S3: Relative proliferative capacity of SCCOHT-1, BIN-67, NIH:OVCAR-3 and SK-OV-3 cells was determined in 24-well plates using the trypan blue exclusion test between 24 h to 72 h. Data represent the mean ± s.d. of 3 independent experiments.

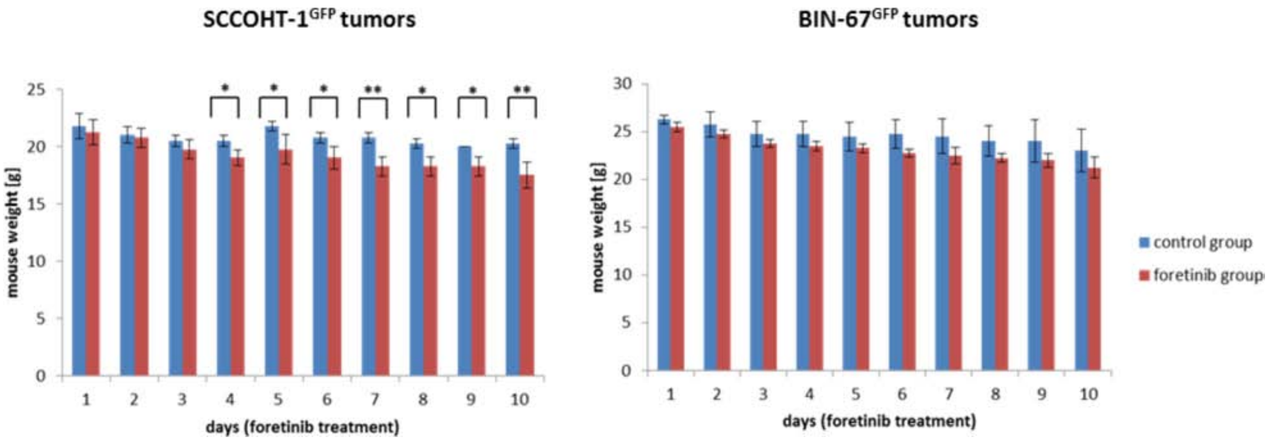

Supplementary Figure S4: Body weight was determined in mice carrying SCCOHT-1^{GFP} (left panel) and BIN-67^{GFP} (right panel) tumor xenografts receiving control treatment compared to foretinib treatment over 10 days. Data represent the mean ± s.d. of 3 animals in each experiments. Statistical analysis was performed by unpaired Student's t-test (*$P < 0.05$; **$P < 0.01$).

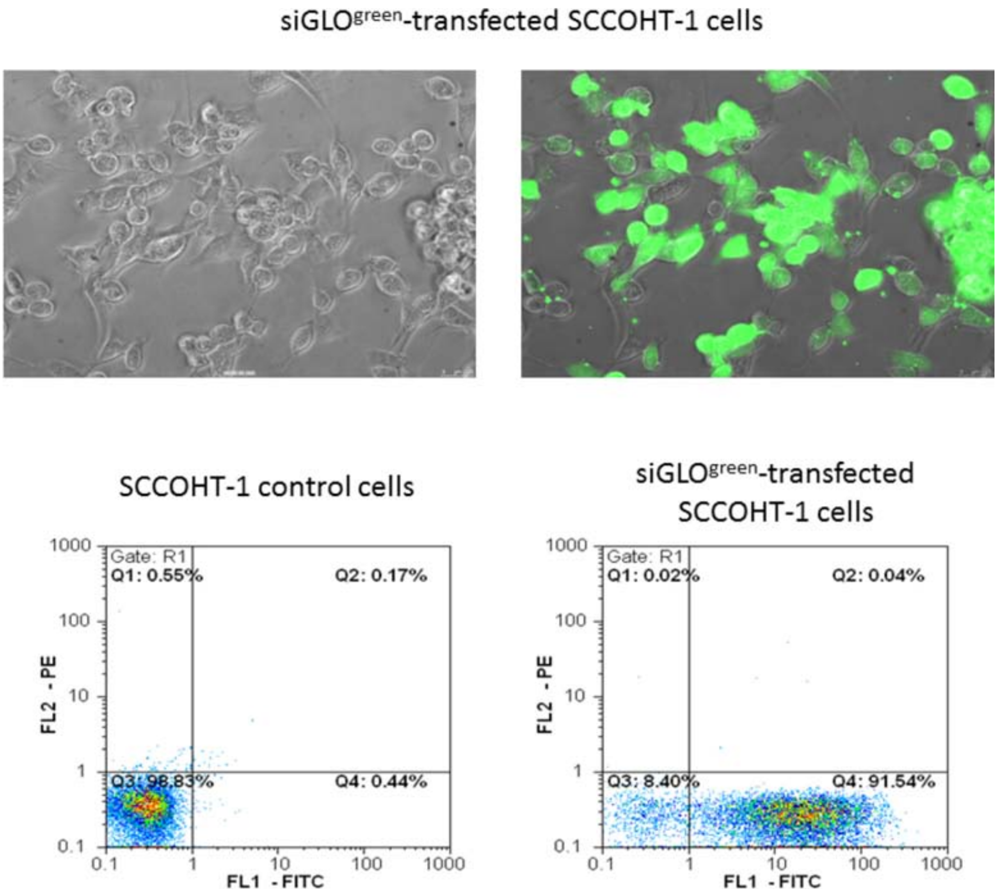

Supplementary Figure S5: Transfection efficiency of SCCOHT-1 cells with 25 nM siGLO^{green} was evaluated by microscopy (upper panels) and by flow cytometry (lower panels) 24h after transfection.

STR fragment	SCCOHT-1	BIN-67
Penta D A1	12	9
Penta D A2	15	9
Penta E A1	10	14
Penta E A2	13	16
AMEL A1	X-chr	X-chr
AMEL A2	X-chr	X-chr
CSF1PO A1	10	10
CSF1PO A2	11	12
D13S317 A1	8	10
D13S317 A2	12	12
D16S539 A1	9	12
D16S539 A2	13	12
D18S51 A1	13	13
D18S51 A2	13	19
D3S1358 A1	15	15
D3S1358 A2	17	19
D7S820 A1	10	9
D7S820 A2	11	9
D8S1179 A1	13	14
D8S1179 A2	14	14
TH01 A1	7	6
TH01 A2	8	9.3
TPOX A1	8	11
TPOX A2	9	11

Supplementary Figure S6: Short tandem repeat (STR) fragment analysis using the GenomeLab human STR primer set was performed for SCCOHT-1 and BIN-67 cell populations. Data from different culture periods of SCCOHT-1 (n = 3), respectively, confirmed reproducible STR patterns for each cell type.
